# Cochlear Implantation Outcomes in Patients With *OTOF* Mutations

**DOI:** 10.3389/fnins.2020.00447

**Published:** 2020-05-21

**Authors:** Dandan Zheng, Xiao Liu

**Affiliations:** Department of Otorhinolaryngology, 2nd Affiliated Hospital, School of Medicine, Zhejiang University, Hangzhou, China

**Keywords:** auditory neuropathy, cochlear implantation, *OTOF*, rehabilitation, outcomes

## Abstract

Auditory neuropathy is a special type of hearing loss caused by dysfunction of the synapse of the inner hair cells, the auditory nerve, and/or the auditory nerve itself. For patients with auditory neuropathy who have severe to profound hearing loss or failed auditory skills development with hearing-aids, cochlear implantation (CI) serves as the only possible effective treatment. It is accepted that the exact sites of lesion causing auditory neuropathy determine the CI performance. Mutations in the *OTOF* gene were the first identified and the most common cause of congenital auditory neuropathy. The site of lesion in patients with auditory neuropathy caused by biallelic *OTOF* mutations (*OTOF*-related auditory neuropathy) is presumed to be presynaptic, leaving auditory nerve function intact. Thus, *OTOF*-related auditory neuropathy is expected to have good CI performances. In this review, we describe the CI outcomes in patients with *OTOF* mutations. We will focus on whether biallelic *OTOF* mutations are ideal indications for CI in patients with auditory neuropathy. Also, the factors that may still influence the CI outcomes in patients with *OTOF* mutations are discussed.

## Introduction

Auditory neuropathy is a type of hearing loss caused by dysfunction of the synapse of the inner hair cells, the auditory nerve, and/or the auditory nerve itself (Hayes and Sininger, 2008). Individuals with auditory neuropathy typically show normal or near-normal otoacoustic emission (OAE) or cochlear microphonics (CM), but absent or abnormal auditory brainstem response (ABR) and/or middle ear muscle reflexes, usually accompanied by poor speech discrimination scores and poor understanding (Starr et al., 1996). For hearing rehabilitation in patients with auditory neuropathy, both cochlear implantation (CI) and wearing hearing-aids (HA) are options. However, in patients who have failed auditory skills development with HA or who are with severe to profound hearing loss, CI is considered the only possible effective treatment (Yawn et al., 2019).

As the transmission of the signal from electrical stimulation of the spiral ganglion provided by the cochlear implants could be affected, it had been thought that the CI outcomes were relatively poor in patients with auditory neuropathy (Starr et al., 1996). Recently, however, studies showed that CI could help to develop auditory skill in some of the patients with auditory neuropathy, yet the benefits were uncertain due to a wide range of etiologies (Rance et al., 1999; Berlin et al., 2010; Humphriss et al., 2013). The cause of auditory neuropathy includes loss of inner hair cells (IHCs) or IHC ribbon synapses, impaired synaptic transmission to spiral ganglion neurons (SGNs), and disrupted propagation of auditory information along the auditory nerve (Moser and Starr, 2016). It is obvious that the exact sites of lesion causing auditory neuropathy determine the CI performance. That is, lesions located in the membranous labyrinth (presynaptic) are associated with good CI performance, while the lesions in the auditory nerve itself (postsynaptic) are not (Eppsteiner et al., 2012).

In the last two decades, genetic defects have been proved that can cause auditory neuropathy [reviewed in Moser and Starr (2016)]. Among these genetic defects, mutations in *OTOF* gene (MIM# 603681) were the first identified and the most common cause of congenital auditory neuropathy (Rodriguez-Ballesteros et al., 2003; Varga et al., 2003; Rodriguez-Ballesteros et al., 2008; Zhang Q. J. et al., 2016). Otoferlin, encoded by the *OTOF* gene, plays an essential role in vesicle releasing and replenishing at the auditory ribbon synapses between IHCs and SGNs (Roux et al., 2006; Pangrsic et al., 2010). Mutations of *OTOF* lead to a reduction of synaptic vesicle exocytosis at ribbon synapse (Roux et al., 2006; Pangrsic et al., 2010; Michalski et al., 2017). Therefore, the site of lesion in auditory neuropathy patients with biallelic *OTOF* mutations (*OTOF*-related auditory neuropathy) is presumed to be presynaptic, and auditory nerve function is assumed to be intact. Theoretically, *OTOF* gene mutations are associated with good CI performances. Indeed, several studies did report “excellent” CI outcomes in patients with *OTOF* mutations. Nevertheless, the evidence that biallelic *OTOF* mutations are associated with good CI outcomes is not yet sufficient due to the small number (*n* ≤ 10) of subjects of these studies.

In this mini review, we will focus on the CI outcomes in patients with *OTOF* mutations. The main goal is to confirm whether biallelic *OTOF* mutations are ideal indications for CI in patients with auditory neuropathy. Also, the factors that may influence the CI outcomes are discussed.

## Methods of Literature Search

**FIGURE 1:**
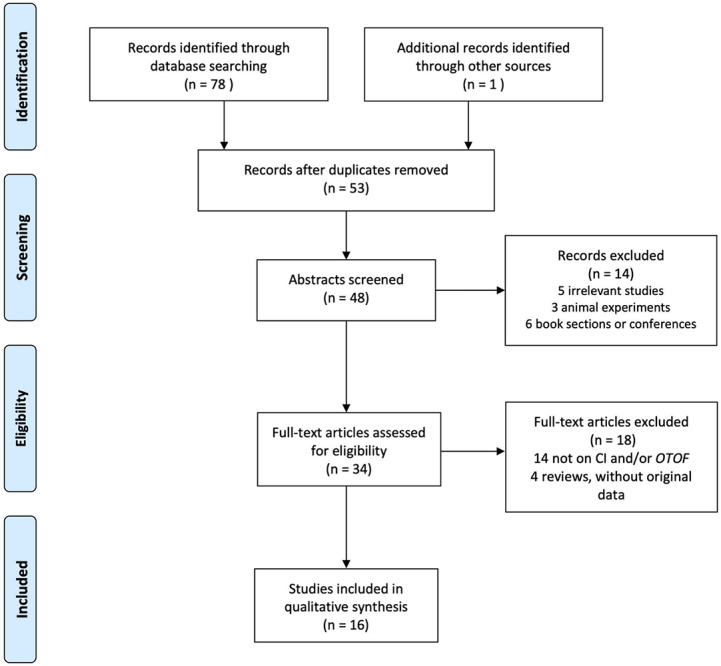
PRISMA flow diagram.

Following Preferred Reporting Items for Systematic Reviews and Meta-Analysis (PRISMA) guidelines (Liberati et al., 2009; Moher et al., 2009), the databases PubMed, Web of Science, and the Cochrane Library were searched for relevant articles published between April 1999 and March 2020. The following search strategy was used to identify eligible studies: (‘*OTOF*’ OR “otoferlin”) AND (‘cochlear implant’ OR ‘cochlear implantation’ OR ‘CI’). All publications were searched and screened by two individuals independently. Additional articles were identified by manually searching known articles. Only full-text, peer-reviewed articles written in English were considered for inclusion. The exclusion of irrelevant studies, animal experiments, and book sections or conferences was made by screening titles and abstracts of the articles. The inclusion criteria and selection were performed through the reading of the full text. The included studies are required to report original CI outcomes in patients with *OTOF* mutations. The flow diagram is shown in Figure 1.

## “Excellent” CI Outcomes in Patients With *OTOF* Mutations

CI for *OTOF*-related auditory neuropathy was first reported in 2003 by Rodriguez-Ballesteros et al. (2003). Ten subjects who met the diagnostic criteria of auditory neuropathy (TEOAEs were present, while ABRs were absent) underwent CI in the study (Rodriguez-Ballesteros et al., 2003). Despite no quantitative indicators, the results of CI in all the 10 subjects were considered to be “successful” (Rodriguez-Ballesteros et al., 2003). Subsequently, CI outcomes were assessed quantitatively in several other case reports and series (Loundon et al., 2005; Rouillon et al., 2006; Runge et al., 2013; Zhang et al., 2013; Santarelli et al., 2015; Miyagawa et al., 2016; Zhang Q. et al., 2016; Park et al., 2017; Chen et al., 2018; Hosoya et al., 2018). All of these studies showed improvement of sound perception and speech recognition with cochlear implants. Most recently, two studies of larger sample sizes (*n* = 10) reviewed the CI outcomes in patients with *OTOF*-related auditory neuropathy, and the results were compared with typical sensorineural hearing loss (SNHL) (Kim et al., 2018; Wu et al., 2018). To the best of our knowledge, CI outcomes have only been reported in approximately 60 patients with *OTOF* mutations (detailed in Table 1, some cases may be shared among studies). Remarkably, almost all of the patients with *OTOF* mutations developed great skills in sound perception and/or speech recognition after CI.

**TABLE 1:** Detailed cochlear implantation performances in patients with *OTOF* mutations.

Author	Year	No. of patients	Age at HL diagnosed	Age at first CI mean(rang)	Follow up duration	CI performances
Rodriguez-Ballesteros et al., 2003	2003	10	–	–	–	The results of CI were successful in terms of sound detection and communication skills.
Loundon et al., 2005	2005	1	10 m	∼4 y	12 m	**PTA (mean of 250–2000 Hz):** 45dB (vs. 75dB with HA) **Speech perception:** 100% (vs. 0% with HA); **IT-MAIS:** 31/40 (vs. 4/40 with HA); **NRT:** good responses on the tested electrodes.
Rouillon et al., 2006	2006	2*	10 m 22 m	4 y 25 m	18 m 36 m	**PTA (mean of 250–2000 Hz):** 37dB and 45dB (vs. 75dB and 75dB with HA); **Closed-set sentences:** 100%; **Open-set words and sentences:** 45–100%; **MAIS:** 40/40 and 31/40 (vs. 4/40 and 4/40 with HA); **Nottingham scale:** grade 4 and 2; **NRT:** all the electrodes with positive responses.
Chiu et al., 2010	2010	3	6 m 6 m 1 y	–	>1 y	A preliminary evaluation of the speech perception performance revealed excellent outcomes in all three patients, comparable to cochlear implantees with *OTOF* mutations Rodriguez-Ballesteros et al., 2003.
Zadro et al., 2010	2010	3	13 m 2 y 30 m	46 m 32 m 36 m	24 m 24 m 12 m	The patients showed awareness to speech sounds, and hearing perceptive abilities achieved the identification level.
Wu et al., 2011	2011	1	–	6 y	3 y	**CAP:** 7 at 3 years.
Runge et al., 2013	2013	2 (siblings)	0.5 9 m	18 m 16 m	3 y	**Speech recognition thresholds:** 44dB in quiet, 52dB in noise, and 65dB in quiet, 70dB in noise; **Lexical Neighborhood Test Easy:** 80% and 44% correct; **ECAP recovery:** one patient had a higher recovery exponent than the average of the pediatric and adult subjects; one had a recovery exponent within the average range.
Zhang et al., 2013	2013	1	12 m	20 m	∼3 y	**PTA (mean of 500–4000 Hz):** 25dB; **NRI:** waveform testing was within normal limits.
Santarelli et al., 2015	2015	6	4 m–2 y	2.1 (1–4)y	1–1.5 y	**Open-set disyllable recognition test:** 90–100%; **ECAP:** increasing stimulation levels resulted in a higher amplitude and a slight decrease in the latency.
Miyagawa et al., 2016	2016	1	–	–	12 m	**LittlEARS auditory questionnaire:** 0, 9, 24, and 30 at 0, 3, 6, and 12 months after CI
Zhang Q. et al., 2016	2016	1	30 m	4 y	2 y	**PTA (mean of 500–4000 Hz):** 37.5 dB; **Open-set words recognition:** 93%; **Open-set sentences recognition:** 98%.
Park et al., 2017	2017	5	–	–	36 m	**CAP:** 6–7 at 24 m in early implantees (age < 24 m), and 3–4 at 24 m in late implantees (age > 24 m).
Chen et al., 2018	2018	1	18 m	4.5 y	24 m	**PTA (mean of 500–4000 Hz):** 31.25dB at 18 m, 30dB at 24 m; **Open-set words:** 90% at 18 m, 95% at 24 m.
Hosoya et al., 2018	2018	4	–	27.8 (21–40) m	–	**CAP:** no significant difference among the patients with *OTOF, GJB2, SLC26A4* mutations and CMV infection; **EABR:** longer wave V, wave III, and Wave III–Wave V latencies.
Kim et al., 2018	2018	10^†^	–	19.2 (13–26) m	36 m	**CAP:** 4–5 at 12 m, 4–7 at 25 m, 7 at 36 m.
						More rapid improvement in early implantees (age ≤ 18 m) than late implantees (age > 18 m).
Wu et al., 2018	2018	10	–	2.9 (1–5.6)y	3 m–5 y	**Speech discrimination score:** 77.5 ± 37.1% at 3 years; **CAP and SIR:** no significant difference among the patients with *OTOF, GJB2* and *SLC26A4* mutations. **NRT and NRI:** all 10 patients revealed robust ECAPs.

*One case may be shared with Loundon et al. (2005); †some cases may be shared with Park et al. (2017); m: month(s); y: year(s); PTA: pure tone audiometry; CAP: categories of auditory performance; CMV: cytomegalovirus; CI: cochlear implant(s); EABR: electrically evoked auditory brainstem responses; ECAP: electrically evoked compound action potential; HA: hearing aid(s); HL: hearing loss; IT-MAIS: infant-toddler meaningful auditory integration scale; MAIS: meaningful auditory integration scale; NRI: neural response imaging; NRT: neural response telemetry; SIR: speech intelligibility rating.

### Sound Perception After CI

Perceiving sound is the initial step and a prerequisite for hearing rehabilitation with CI. The ability to perceive sound after CI was evaluated by audiometry. Loundon et al. (2005) and Rouillon et al. (2006) found that mean pure tone thresholds of 250, 500, 1000 and 2000 Hz in the patients with *OTOF*-related auditory neuropathy improved from 75 dB with HA to 37 and 45 dB with the cochlear implants after 1–1.5 years of rehabilitation. Similarly, Zhang et al. (2013), Zhang Q. et al. (2016), and Chen et al. (2018) found that the mean pure tone thresholds of 500, 1000, 2000 and 4000 Hz received 25 to 37.5 dB with cochlear implants after more than 2-year of rehabilitation. Although it is still unknown whether the audiometric thresholds would continue to improve with the extension of the rehabilitation time, the available data have shown that the sound perception in patients with *OTOF* mutations can be significantly improved by CI.

### Speech Recognition After CI

Being able to understand speech is one of the main purposes of CI rehabilitation. Thus, speech recognition is a direct indicator of CI outcomes evaluation. Objective indicators, such as speech perception testing and speech recognition thresholds, showed that all the patients with *OTOF* mutations benefited from CI. In terms of speech perception, most of the patients with *OTOF* mutations got a ≥90% score in closed-set or open-set perception (detailed in Table 1; Loundon et al., 2005; Rouillon et al., 2006; Santarelli et al., 2015; Zhang Q. et al., 2016; Chen et al., 2018). More recently, Wu et al. (2018) reviewed 10 cases of *OTOF*-related auditory neuropathy and found that the speech discrimination score received 77.5 ± 37.1% at 3 years. In speech recognition thresholds, Runge et al. (2013) reported 2 cases (siblings) of *OTOF*-related auditory neuropathy and found that the thresholds were 44 and 65 dB in quiet, 52 and 70 dB in noise, respectively.

In addition, scales, such as meaningful auditory integration scale/infant-toddler meaningful auditory integration scale (MAIS/IT-MAIS), categories of auditory performance (CAP) and speech intelligibility rating (SIR) are widely used to evaluate the CI performances. In the case reports by Loundon et al. (2005) and Rouillon et al. (2006), the MAIS/IT-MAIS scores increased from 4/40 and 4/40 with HA to 40/40 and 31/40 with cochlear implants. In terms of CAP and SIR, patients with *OTOF* mutations showed rapid improvement of scores after CI (Wu et al., 2018). The CAP scores reached 6/6–7/7 during the 2–3 year follow up (Wu et al., 2011; Park et al., 2017; Kim et al., 2018). Moreover, studies by Hosoya et al. (2018) and Wu et al. (2018) showed that there was no significant difference in CAP or SIR scores among patients with *OTOF*, *GJB2, SLC26A4* mutations or cytomegalovirus infections.

According to the literature, *OTOF*-related auditory neuropathy is associated with excellent CI outcomes. Patients with this type of auditory neuropathy can not only “hear” the sound, but also “understand” the speech well with the help of cochlear implants. Unlike other types of auditory neuropathy, the CI performances of the patients with biallelic *OTOF* mutations are predictable and comparable to those of “typical” SNHL. A detection of *OTOF* mutations can help in accurately localizing the site of lesion and informing therapy-related clinical decision making in patients with auditory neuropathy.

## Factors That May Influence CI Outcomes in *OTOF*-Related Auditory Neuropathy

Although all studies were in coherence with that the auditory neuropathy caused by *OTOF* mutations tend to have good CI outcomes, individual variations still exist among cases. For example, Runge et al. (2013) reported a sibling pair diagnosed with *OTOF*-related auditory neuropathy. The genotypes of these siblings were the same, but the speech perception performance differed between the siblings. In another study, Park et al. (2017) followed up four subjects with *OTOF*-related auditory neuropathy who underwent CI and found that the CAP scales ranged from 3 to 7 at 24 months post-CI. Due to the limited number of cases, it is impossible to ascertain what is the exact factors that may influence the CI outcomes in *OTOF*-related auditory neuropathy. However, clues may be provided by these cases.

### Age at Implantation

Earlier implantation is associated with better CI outcomes in patients with *OTOF* mutations. It has been widely accepted that early implantation is good for CI outcomes in patients with typical SNHL (Niparko et al., 2010; Black et al., 2011; Panda et al., 2019). 0 to 3.5 years of age is considered a critical period for first language acquisition, and implantation after that period tends to have poorer outcomes (Sharma et al., 2002; Kral and Sharma, 2012). For patients with auditory neuropathy, the critical period seems to be narrower than those with typical severe to profound SNHL. That is, patients with auditory neuropathy who undergo cochlear implantation before the age of 2 years may have better auditory outcomes than those after the age of 2 years (Cardon and Sharma, 2013; Liu et al., 2014). This could be explained by that the disordered pattern of neural input to the cortex, as a result of auditory nerve dys-synchrony in patients with auditory neuropathy have negative effects on central auditory maturation (Cardon and Sharma, 2013; Sharma and Cardon, 2015).

As mentioned before, the site of lesion in patients with *OTOF* mutations is assumed to be presynaptic (Roux et al., 2006; Pangrsic et al., 2010). Thus, the pathological mechanism of *OTOF*-related auditory neuropathy is thought to be more like a typical SNHL but not an auditory neuropathy. However, Park et al. (2017) found that early (<24 months) implantees experienced notably better outcomes than late (>24 months) implantees. Similarly, Kim et al. (2018) found that the early (≤18 months) implantees had better outcomes than the late (>18 months) implantees at 6 months after CI. Furthermore, listening skills improved more rapidly in early implantees than late implantees (Kim et al., 2018). All the above suggest that patients with auditory neuropathy caused by mutations of *OTOF* seemed to be more affected by delayed implantation than those with typical SNHL that caused by mutations of *SLC26A4* and *GJB2* (Park et al., 2017).

The above results still require further confirmation as the sample size of existing comparative studies were small (*n* ≤ 10). Besides, late implantation (6 years of age) reported by Wu et al. (2011) also showed with a “good” CI outcome, which implied that late implantation was still beneficial for some of the patients. However, from a clinicians’ point of view, most of the patients with *OTOF* mutations did not experience spontaneous recovery of auditory performance, it was more likely that early implantation (<24 years) could achieve optimal CI performances (Wu et al., 2018).

### The Integrity of the Auditory Nerve Function

The integrity of auditory nerve function is a key determinant of CI performances, especially for patients with auditory neuropathy. The auditory nerve function in *OTOF*-related auditory neuropathy is usually presumed intact. This assertion was supported by testing the neural responses of SGNs. Neural responses eluted by CI are objective indicators for evaluating the ability of the auditory pathway to receive, transmit and process complex electrical signals. By testing electrically evoked compound action potentials (ECAPs) or electrically evoked auditory brainstem responses (EABRs), the auditory nerve has been proved to have “good response” to cochlear implant stimuli (Loundon et al., 2005; Rouillon et al., 2006; Runge et al., 2013; Zhang et al., 2013; Santarelli et al., 2015; Wu et al., 2018).

However, some other studies might challenge the above view. Runge et al. (2013) quantitatively analyzed ECAP recovery rates in the sibling pair with *OTOF*-related auditory neuropathy. Though with the same genotype, one sibling had a recovery exponent within the average range of SNHL, while the other one had a more than one standard deviation (SD) higher recovery exponent than the average range of SNHL. Hosoya et al. (2018) tested the EABRs in patients with congenital hearing loss and found that the Wave III, Wave V, and Wave III–Wave V latencies were significantly longer in patients with *OTOF* mutations than those in SNHL. These two studies implicated that *OTOF* mutations might affect the more central auditory pathway beyond the synapse between the IHCs and SGNs. In addition, neurological and/or central pathologies should not necessarily be ruled out even when a patient was diagnosed with *OTOF*-related auditory neuropathy.

### Genotypes of the OTOF Gene

As mutations of the *OTOF* gene are the cause of the disease, it is reasonable to speculate whether CI outcomes are associated with distinct genotypes. To date, more than 130 variants in the *OTOF* gene have been implicated pathogenic or likely pathogenic^1^. Although patients who underwent CI showed a high frequency of p.Gln829^∗^ in European (Rodriguez-Ballesteros et al., 2003; Loundon et al., 2005; Rouillon et al., 2006), p.Glu1700Gln in Chinese (Chiu et al., 2010; Wu et al., 2011; Wu et al., 2018) and p.Arg1939Glu in Korean (Park et al., 2017; Kim et al., 2018) population, more than 30 different *OTOF* mutations have been detected in the CI recipients (listed in Supplementary Table 1). Due to the different methods of CI performance evaluation and the variety of genotypes, it is impracticable to compare CI outcomes among patients with different genotypes directly.

Nevertheless, it is still viable to investigate whether there is a correlation between mutation types and CI outcomes. Otoferlin has six C2 domains, one Fer-like structure and one transmembrane domain (TMD). Nonsense mutations (like p.Gln829^∗^) usually lead to the loss of C2 domain(s) and the TMD, and result in a complete loss of otoferlin function (Migliosi et al., 2002), while missense mutations (like p.Glu1700Gln and p.Arg1939Glu) might affect only one C2 domain, the TMD, or neither of them (Varga et al., 2003; Chiu et al., 2010; Zhang Q. J. et al., 2016). Based on the available data, all patients with homozygous p.Gln829^∗^, p.Glu1700Gln or p.Arg1939Glu revealed excellent outcomes in sound perception and/or speech recognition (Rodriguez-Ballesteros et al., 2003; Chiu et al., 2010; Wu et al., 2011; Kim et al., 2018). Therefore, there is insufficient evidence that CI outcomes are correlated with distinct *OTOF* genotypes.

### Recommendations Regarding the CI in *OTOF*-Related Auditory Neuropathy

As most of the patients with *OTOF*-related auditory neuropathy presented a phenotype of stable, severe to profound non-syndromic hearing loss (Zhang Q. J. et al., 2016), it seems that CI is the optimal and the only defective treatment option. However, CI may not be suitable for all patients with *OTOF* mutations. Firstly, the confirmation of *OTOF*-related auditory neuropathy could be challenging. There is no well-accepted hotspot mutation in *OTOF* except p.Gln829^∗^ in Spanish (Migliosi et al., 2002), and the number of novel *OTOF* mutations is growing, but most of the mutations lack functional studies. In a patient with *OTOF* mutations, it is difficult to confirm that the hearing loss is caused by otoferlin (*OTOF*) deficiency and rule out other causes. Secondly, some of the patients with *OTOF* mutations manifested as temperature sensitive auditory neuropathy (TS-AN), i.e. the hearing thresholds fluctuate with a variation of core body temperature and may improve with age (Zhang Q. et al., 2016). In these cases, HA could be an effective treatment. Thus, HA trials are still recommended in the patients with mild to moderate fluctuating hearing loss, but once a patient was identified with severe to profound hearing loss or fail to develop age-appropriate language skills, CI would be considered. Due to a possible narrower critical period for CI, earlier (<24 months) implantation is recommended (Cardon and Sharma, 2013). Before the operation, clinical manifestations, molecular test results, and the auditory nerve functions should be comprehensively assessed to exclude TS-AN and auditory neuropathy caused by other reasons.

## Conclusion

The existing literature consistently revealed that patients with *OTOF* mutations are associated with excellent CI performance in both sound perception and speech recognition. Genetic analysis of *OTOF* can provide great help in localizing the site of lesion and informing therapy-related clinical decision making. Auditory neuropathy with biallelic *OTOF* mutations is an ideal surgical indication for CI. Notably, compared with typical SNHL, a narrower critical period for CI was implied in patients with *OTOF* mutations. Thus, once diagnosed as an *OTOF*-related auditory neuropathy with severe to profound hearing loss, early implantation is recommended. In addition, although the auditory nerve function is normal in most of the patients with *OTOF* mutations, neurological and/or central pathologies should not be ruled out in these cases. There is no evidence that CI outcomes are correlated with distinct *OTOF* genotypes. Compared with typical SNHL, the sample size of the studies on CI outcomes in patients with *OTOF* mutations is small. Future studies with larger sample sizes are required to confirm this conclusion.

## Author Contributions

XL conceived the review. DZ participated in drafting the manuscript. Both authors wrote the manuscript.

## Conflict of Interest

The authors declare that the research was conducted in the absence of any commercial or financial relationships that could be construed as a potential conflict of interest.

## Footnotes

1 http://deafnessvariationdatabase.org/gene_page/OTOF
